# Resting myocardial perfusion quantification with CMR arterial spin labeling at 1.5 T and 3.0 T

**DOI:** 10.1186/1532-429X-10-53

**Published:** 2008-11-17

**Authors:** Benjamin E Northrup, Kyle S McCommis, Haosen Zhang, Shuddhadeb Ray, Pamela K Woodard, Robert J Gropler, Jie Zheng

**Affiliations:** 1 Mallinckrodt Institute of Radiology, Washington University School of Medicine, St. Louis, Missouri, USA; 2 Dartmouth Medical School, Hanover, New Hampshire, USA; 3 University of Kansas School of Medicine, Kansas City, Kansas, USA

## Abstract

**Background:**

The magnetic resonance technique of arterial spin labeling (ASL) allows myocardial perfusion to be quantified without the use of a contrast agent. This study aimed to use a modified ASL technique and *T*_1 _regression algorithm, previously validated in canine models, to calculate myocardial blood flow (MBF) in normal human subjects and to compare the accuracy and repeatability of this calculation at 1.5 T and 3.0 T. A computer simulation was performed and compared with experimental findings.

**Results:**

Eight subjects were imaged, with scans at 3.0 T showing significantly higher *T*_1 _values (*P *< 0.001) and signal-to-noise ratios (SNR) (*P *< 0.002) than scans at 1.5 T. The average MBF was found to be 0.990 ± 0.302 mL/g/min at 1.5 T and 1.058 ± 0.187 mL/g/min at 3.0 T. The repeatability at 3.0 T was improved 43% over that at 1.5 T, although no statistically significant difference was found between the two field strengths. In the simulation, the accuracy and the repeatability of the MBF calculations were 61% and 38% higher, respectively, at 3.0 T than at 1.5 T, but no statistically significant differences were observed. There were no significant differences between the myocardial perfusion data sets obtained from the two independent observers. Additionally, there was a trend toward less variation in the perfusion data from the two observers at 3.0 T as compared to 1.5 T.

**Conclusion:**

This suggests that this ASL technique can be used, preferably at 3.0 T, to quantify myocardial perfusion in humans and with further development could be useful in the clinical setting as an alternative method of perfusion analysis.

## Background

Myocardial blood flow (MBF), defined as the rate at which blood enters the myocardial microvasculature through the coronary artery network, is an important indicator of myocardial perfusion changes, as seen in myocardial stress and ischemic heart disease [1-3]. The gold standard for human myocardial perfusion analysis is positron emission tomography (PET), but this technique is marred by limited spatial resolution, high cost, limited availability, and patient radiation exposure [4]. Additionally, first-pass perfusion cardiovascular magnetic resonance (CMR) would allow efficient measurement of myocardial perfusion, but the necessity of a gadolinium-based contrast agent would limit this technique in terms of repetition in a single CMR examination and use in the renal failure patient. However, arterial spin labeling (ASL), through measurement of myocardial and blood *T*_1_, is capable of measuring myocardial perfusion multiple times in a single CMR examination [2,5]. ASL uses arterial water as an endogenous tracer and can be implemented through a number of techniques. The method used in this work is based on the flow-sensitive alternating inversion recovery (FAIR) scheme. In FAIR, two sets of inversion recovery (IR) images are acquired: a slice-selective inversion and a nonselective inversion [6]. After the slice-selective inversion, non-inverted proton spins from arterial water flow into the imaging slice and exchange with tissue water, resulting in *T*_1 _shortening in the tissue of interest.

Previously, two studies involving the rat heart in various states (resting, hyperemic, and during post-infarction remodeling) have validated ASL against the previously established technique of myocardial perfusion quantification using microspheres [7,8]. Furthermore, ASL has been validated with both the microsphere technique and first-pass perfusion imaging in a different study using rats [9]. Notably, ASL has been shown to be successful in its application to humans with ischemic heart disease and in the determination of perfusion reserve, a clinically useful metric, in a study that included patients with suspected coronary artery disease [10].

From ASL sequences, myocardial perfusion can be quantified by calculating MBF using the following equation [5]:

(1) $$MBF=\lambda \frac{T_{1,GS}}{T_{1,Blood}}(\frac{1}{T_{1,SS}}-\frac{1}{T_{1,GS}})\;$$

where λ is the constant blood-tissue coefficient of water, and λ = 0.92 mL/g for blood-perfused myocardial tissue [11]; *T*_1, GS _is the *T*_1 _of the myocardium after the nonselective IR pulse; *T*_1, Blood _is the average *T*_1 _of the left ventricular blood pool; and *T*_1, SS _is the *T*_1 _of the myocardium after the slice-selective IR pulse. This equation assumes identical spatial slice profiles of the slice-selective IR pulse and the slice-selective excitation pulse, a two-compartment model (consisting of the intravascular capillary blood and the extravascular tissue) in which magnetization is homogeneous in each compartment, and rapid exchange of water between the two compartments. The accuracy of the myocardial perfusion rate determined by this equation is highly dependent on both the measured *T*_1 _values and the ASL technique used to obtain them. Recently, we have proposed a modified ASL technique and *T*_1 _regression algorithm that has proved reliable in assessing MBF in canine models [2,12].

In recent years, 3.0 Tesla (T) magnetic resonance imaging systems have been used increasingly in the clinical setting. The primary advantage of 3.0 T over 1.5 T systems is the anticipated, but less than, twofold increase in signal-to-noise ratio (SNR) [13-15]. Though it is the intrinsically longer *T*_1 _relaxation times observed at 3.0 T that lead to this lower-than-expected SNR increase in *T*_1_-weighted imaging, 3.0 T systems still demonstrate a significantly higher SNR than that of 1.5 T, and thus serve as a more reliable platform for the calculation of *T*_1 _[16,17]. Additionally, the intrinsically longer *T*_1 _relaxation times grant 3.0 T greater sensitivity to *T*_1 _changes.

Accordingly, the primary aim of this study was to compare the accuracy and repeatability of myocardial perfusion measurements by the use of a recently modified ASL technique at 3.0 T to those at 1.5 T in resting healthy human subjects [2].

## Methods

### Imaging protocol

A total of 8 subjects (5 male, 3 female, mean age 21.9 ± 2.0 years) were recruited. All subjects had no known history of cardiovascular disease and were in the resting state for both CMR examinations. Each patient underwent two consecutive same-day imaging sessions, one on a 1.5 T whole-body Sonata system (Siemens Medical Solutions, Erlangen, Germany) and one on a 3.0 T whole-body Magnetom Trio system (Siemens Medical Solutions, Erlangen, Germany). Both systems were equipped with a fast gradient system (maximal gradient strength = 40 mT/m; maximal slew rate = 200 mT/m per millisecond). A four-element phased array coil was used for signal reception and a body coil was used for transmission. For electrocardiogram (ECG) monitoring and pulse sequence triggering, a three-lead patch was attached to the chest of each subject. This allowed for the acquisition of images at mid-diastole, the time of least cardiac contractile motion. To eliminate respiratory motion artifacts, the subjects were instructed to hold their breath for the duration of each series of acquisitions [18].

**Figure 1:**
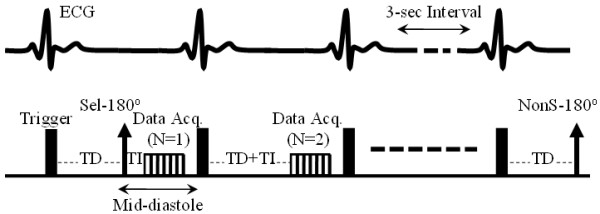
A schematic diagram of the sequence that was used to obtain the data necessary for myocardial perfusion quantification, highlighting ECG triggering and the 3 second idle time between the two IR acquisitions.

A scout image was first obtained to localize a short axis view of the left ventricle (LV) of each subject's heart. Next, a cine acquisition with a segmented steady-state free precession (SSFP) sequence was performed to determine the mid-diastolic period in the cardiac cycle [19]. *T*_1_-weighted images were then acquired using a recently developed single-shot gradient-echo (GE)-based ASL sequence with an adiabatic hyperbolic secant IR pulse [2]. At both main magnetic field [*B*_0_] strengths, the previous ASL sequence was modified by combining nonselective (NonS) and slice-selective (Sel) prepared sequences into one series. This allowed for the reduction of inter-scan motion effects and total scanning time. Based on the Look-Locker scheme, the sequence acquired multiple data points along the magnetization recovery time course after each IR pulse. There was a 3-second idle time between the two IR acquisitions to allow more time for the recovery of magnetizations of GE acquisitions. Therefore, the NonS and Sel *T*_1_-weighted signals were acquired sequentially. Data acquisition was ECG-triggered (Figure 1). Each pair of series was repeated three times in order to evaluate the reliability of ASL in perfusion measurement.

### Imaging parameters

On the 1.5 T system, ASL sequence parameters included a TR range of 2.47 to 2.59 ms and a TE range of 1.07 to 1.12 ms. At 3.0 T the TR range was 2.48 to 3.16 ms and the TE range was 1.06 to 1.69 ms. At both *B*_0 _strengths, flip angle = 5°, slice thickness = 8 mm, receive bandwidth = 650 Hz, the number of phase-encoding steps ranged from 64 to 73, and the interpolated matrix size ranged from 128 × 64 to 128 × 73. The first series consisted of 20 to 24 image acquisitions (half obtained with a Sel IR pulse and half obtained with a NonS IR pulse), temporally separated by the RR interval. The average observed RR interval was approximately 800 ms, making the total duration of the series, and thus the breath hold duration, 16 to 19 seconds. The first series was followed immediately by a similar second series of acquisitions (data from both parts were combined in the analysis, described below) with a longer initial inversion time (TI). At 1.5 T, the initial inversion time from the first series (initial TI_1_) ranged from 91 to 99 ms, while that of the second series (initial TI_2_) ranged from 137 to 155 ms. At 3.0 T, the initial TI_1 _ranged from 91 to 122 ms and the initial TI_2 _ranged from 137 to 177 ms. The TI was the time from the middle of the IR pulse to the time when the central k-space data of the GE acquisition was acquired. Thus, the initial TI_1 _was the minimal TI achieved with the defined image matrix size since k-space data was linearly acquired in each *T*_1_-weighted image. Generally, the TI_2 _was approximately 40 ms greater than the TI_1_. This ensured that the IR pulse of the second series, together with the following GE acquisition, remained in mid-diastole [2].

### Data analysis

Two independent observers drew one region of interest (ROI) in the left ventricular blood pool and one in the entire left ventricular myocardial ring. The use of two independent observers facilitated the determination of the inter-observer variability for the calculated MBF values (note that the *T*_1 _and MBF values presented in the results and the tables represent data from a single observer). The ROI in the left ventricular myocardial ring were drawn in the central 50% of the myocardium, in order to avoid artifacts common to the edges. However, the ROI were not drawn over large full-thickness artifacts that were readily visualized, such as air-solid interface magnetic susceptibility artifacts of the lateral wall or blood-pool and myocardial partial volume effects at the septum [20]. Notably, ROI were not drawn over major coronary artery branches so MBF calculations would presumably include data from the level of the myocardial microvasculature only, necessary for this method to accurately quantify myocardial perfusion. Bulk adjustment of a ROI was performed if necessary to correct for positional changes. In addition to this, each image in a pair of series was screened by one observer for motion of any kind, and the pair was given a motion rating on a scale of zero to five (0 = no motion and 5 = severe motion artifacts). Each score was subsequently classified as respiratory, cardiac, or inter-series related motion.

**Figure 2:**
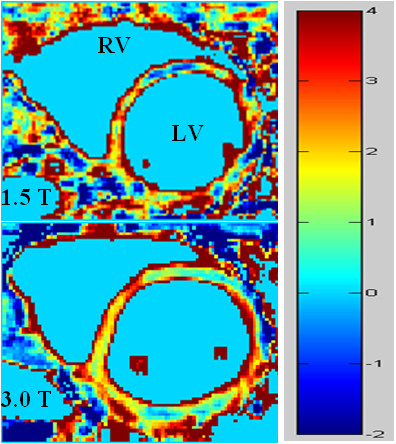
**Perfusion maps at 1.5 T and 3.0 T, **demonstrating a more uniform myocardial perfusion distribution at 3.0 T**. **Each map was created pixel-by-pixel using the corresponding nonselective and slice-selective *T*_1 _maps.

From the absolute values of the signal intensities measured in each ROI, our previously developed MatLab (The MathWorks Co., Natick, MA)-based nonlinear regression algorithm using real clock time for TI was used to calculate *T*_1 _values [2]. Using these values in Eq.[1], MBF was calculated. The average MBF values, as well as the standard deviations (SD), were calculated from all of the pairs of series from each subject at 1.5 T and 3.0 T, respectively. The repeatability was calculated as the ratio of the average SD to the average MBF. Paired comparison of the two field strengths was performed using a paired two-tailed Student's *t *test with a *P *< 0.05 significance level. The strength of agreement between the two sets of observations was determined via MBF comparisons by paired Student's *t *tests and Bland-Altman tests. To demonstrate the capability of perfusion mapping by this method, a myocardial perfusion map was created pixel-by-pixel using corresponding nonselective and slice-selective *T*_1 _maps (Figure 2).

Additionally, SNR was calculated for the entire LV myocardial ring (from both slice-selective and nonselective series) and the LV blood pool (from nonselective series only). This was done by dividing the mean signal intensity (of the LV blood pool or the myocardium) by the standard deviation of the signal intensity of extracorporeal air. In both the experimental and the simulation results (see below), the SNR values from the last image acquired (the image with the longest TI) in each sequence were used.

### Error analysis

To evaluate the effect of SNR on the accuracy and precision of the myocardial perfusion measurements using this ASL method, a computer simulation was performed with the *T*_1 _data sets from the averaged values of volunteers (see Results). Using the following equation (Eq. [12] in reference [2]), signal intensity of S_N _(N is the number of images collected after the IR pulse) can be derived:

(2) $$\frac{S_{N}}{sin\alpha }=M_{0}\left(\text{1}+cos\beta e^{-\tau_{N}/T_{1}}\right)B^{n/2-1}+A^{n/2-1}+A^{n/2}cos\alpha e^{-\tau_{N}/T_{1}}B^{n/2-1}+\frac{S_{N-1}}{sin\alpha }B^{n-1}cos\alpha e^{-\tau_{N}/T_{1}}$$

Where A^n/2-1 ^is defined as $M_{0}(\text{1}-e^{-TR/T_{1}})\frac{\text{1}-{\left(\cos\alpha e^{-TR/T_{1}}\right)}^{n/2-1}}{\text{1}℮\cos\alpha e^{-TR/T_{1}}}$ and B is equal to $\cos\alpha e^{-TR/T_{1}}$, β is the inversion pulse flip angle, α is the RF-train excitation angle, and τ_N _is the time interval between the *(N - 1)th *and *Nth *measurements. This equation was derived from the Bloch Equation to correlate *T*_1_-weighted signal intensities of dynamic images during *T*_1 _recovery, necessary due to the brevity of the RR interval. Using this equation, the saturation effect induced by the multiple data acquisitions between each cardiac cycle can be accounted for in the *T*_1 _calculation.

Random Gaussian noise was then added to the S_N _and SNR was calculated as $\frac{M_{0}\sin\alpha }{Noise}$. Different SNR values were designed and corresponding nonselective and slice-selective myocardial *T*_1 _values were calculated using the nonlinear regression algorithm, as well as non-selective blood *T*_1 _values. MBF values were calculated using Eq. [1] to compare with the true MBF data (data without noise) for accuracy analysis. For the specific *T*_1 _and SNR data sets of the volunteers at 1.5 T and 3.0 T, these procedures were repeated 3 times to calculate the repeatability and aid in the estimation of the accuracy of the means.

## Results

**Figure 3:**
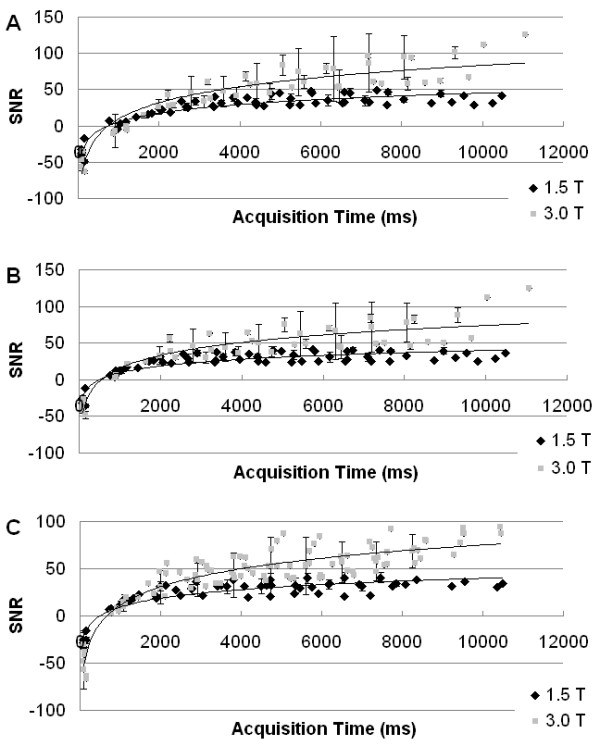
**Normalized dynamic curves of *T*_1 _recovery**, averaged for all subjects: (A) displays data from the LV blood pool (nonselective sequences), (B) displays data from the global LV myocardium from nonselective sequences, and (C) displays data from the global LV myocardium from slice-selective sequences**.** Larger *T*_1 _values are evident at 3.0 T, as is a trend of a greater signal-to-noise ratio (SNR) at most time points.

The individual and overall average *T*_1 _values, calculated from the data from one observer, are shown in Table 1. The average LV blood pool *T*_1_, nonselective myocardial *T*_1_, and slice-selective myocardial *T*_1 _values all showed statistically significant increases from 1.5 T to 3.0 T (*P *< 0.001). Dynamic normalized (to noise) curves of *T*_1 _recovery are demonstrated in Figure 3. A trend of greater SNR at most time points, as well as larger *T*_1 _values, was evident at 3.0 T as compared to 1.5 T.

**Table 1:** The 8 subjects' mean *T*_1 _values (in msec) from Observer 1.

	**1.5 T**			**3.0 T**		
		
Subject	*T*_1, Blood_	*T*_1, GS_	*T*_1, SS_	*T*_1, Blood_	*T*_1, GS_	*T*_1, SS_
1	1743.1 ± 29.5	1003.0 ± 10.9	973.9 ± 11.9	2045.2 ± 25.0	1317.5 ± 5.6	1267.9 ± 13.0
2	1612.6 ± 10.8	1012.1 ± 12.2	987.6 ± 34.2	1856.0 ± 10.0	1306.0 ± 17.9	1268.7 ± 23.6
3	1506.4 ± 24.6	1037.0 ± 1.7	1016.3 ± 8.0	1863.3 ± 39.8	1281.4 ± 7.0	1248.3 ± 11.6
4	1622.0 ± 87.2	839.4 ± 3.2	823.2 ± 2.3	1769.7	1269.7	1219.1
5	1707.2 ± 14.0	1044.0 ± 10.8	1010.3 ± 22.2	1903.7 ± 27.3	1332.1 ± 15.0	1289.3 ± 31.3
6	1589.3 ± 41.1	1046.6 ± 18.6	1014.5 ± 29.9	1889.0 ± 21.8	1318.9 ± 27.9	1274.8 ± 28.1
7	1813.0 ± 89.3	964.7 ± 5.5	915.3 ± 1.4	1860.7 ± 30.6	1253.6 ± 3.9	1200.9 ± 13.4
8	1662.4 ± 117.8	995.3 ± 9.1	971.5 ± 1.2	1793.4 ± 2.5	1287.8 ± 13.1	1240.7 ± 2.3

Mean	1657.4 ± 96.2	992.4 ± 67.9	963.6 ± 65.8	1871.0 ± 85.0	1290.2 ± 33.2	1236.2 ± 55.2

*T*_1, Blood _= *T*_1 _of the left ventricular blood pool; *T*_1, GS _= *T*_1 _of the myocardium after the nonselective IR pulse; *T*_1, SS _= *T*_1 _of the myocardium after the slice-selective IR pulse.

**Figure 4:**
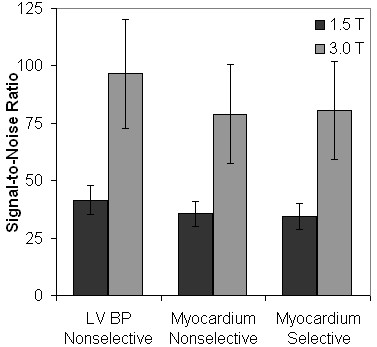
**The average SNR of the LV blood pool (nonselective sequences) and the entire LV myocardial ring (slice-selective and nonselective sequences), calculated from the final acquisition of each sequence.** All average SNR demonstrate statistically significant increases from 1.5 T to 3.0 T (*P *< 0.002). LV BP = Left Ventricular Blood Pool.

The average calculated SNR values from the final acquisition of each sequence are displayed in Figure 4. The average LV blood pool SNR from nonselective sequences (41.56 ± 6.19 at 1.5 T and 96.51 ± 23.57 at 3.0 T), global myocardial SNR from nonselective sequences (35.60 ± 5.43 and 79.01 ± 21.50), and global myocardial SNR from slice-selective sequences (34.54 ± 5.65 and 80.65 ± 21.37) all showed statistically significant increases from 1.5 T to 3.0 T (*P *< 0.002).

The myocardial perfusion data (the average of the MBF values calculated from Eq. [1] for each subject) for the eight subjects, as well as their demographic data and motion ratings are shown in Table 2. Higher standard deviations (SD) of MBF values were observed at 1.5 T in subjects 1, 2, 3, and 6. Higher SD of MBF values were observed at 3.0 T in subjects 5, 7, and 8. In subject 4, the SD could not be compared because only one pair of series of images was acquired at 3.0 T. The average SD at 1.5 T was 0.365, representing a repeatability of 0.37, whereas the SD at 3.0 T was 0.221, representing a repeatability of 0.21, a 43% improvement. In subjects 4, 5, and 6, the 1.5 T scans had higher average motion ratings than the 3.0 T scans. In subjects, 1, 2, 3, 7, and 8, the 3.0 T scans had higher average motion ratings than the 1.5 T scans.

**Table 2:** The 8 subjects' demographic data, scan characteristics, perfusion data, and motion ratings from Observer 1.

		**Pairs of series**		**Average MBF (mL/g/min)**		**Average motion rating per pair of series**			
Subj	Age/Sex	1.5 T	3.0 T	1.5 T	3.0 T	1.5 T	Type	3.0 T	Type
1	22/F	3	3	0.951 ± 0.371	1.060 ± 0.184	0	-	0.33	S
2	20/M	3	3	0.871 ± 0.802	0.874 ± 0.179	1	S	2.67	R
3	23/M	3	2	0.745 ± 0.243	0.789 ± 0.133	0.67	R	1	S, R
4	24/F	2	1	0.678 ± 0.268	1.296	2	R	1	S
5	22/M	3	3	1.088 ± 0.397	0.968 ± 0.438	1.67	S, R	0.67	S, R
6	22/F	3	3	1.102 ± 0.449	1.010 ± 0.043	3.67	S, C	1.67	C
7	18/M	3	3	1.641 ± 0.169	1.303 ± 0.301	1	S, R	2.67	S, R, C
8	24/M	2	2	0.844 ± 0.222	1.169 ± 0.268	0	-	0.5	C

Mean	21.9 ± 2.0			0.990 ± 0.302	1.058 ± 0.187	1.25 ± 1.21		1.31 ± 0.93	

"Pairs of series" denotes each set of back-to-back series. The motion ratings are on a scale of 0 to 5, with 0 representing no detectable motion and 5 representing severe motion artifacts. Subj = subject; S = interseries motion or motion between the pair of series; R = respiratory motion; C = cardiac motion.

**Figure 5:**
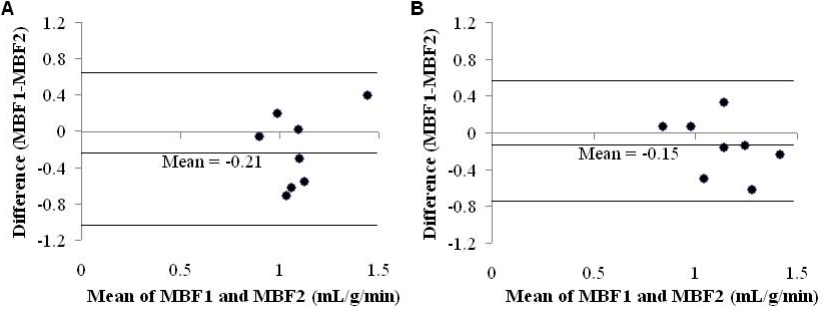
**Bland-Altman plots comparing the myocardial perfusion data obtained by the two independent observers (MBF1 and MBF2) at 1.5 T (A) and 3.0 T (B).** The 3.0 T system demonstrated a trend towards reduced variation (mean = -0.153 and 95% confidence interval of -0.772 to 0.467) in MBF measurements by the two observers, as compared to the 1.5 T system (mean = -0.208 and 95% confidence interval of -1.021 to 0.605). Both axes are in units of mL/g/min.

The average myocardial perfusion rate for all subjects, calculated from the MBF data from one observer, was found to be 0.990 ± 0.302 mL/g/min at 1.5 T and 1.058 ± 0.187 mL/g/min at 3.0 T. This difference between the two field strengths was not found to be statistically significant (*P *= NS). Likewise, the average standard deviations of the MBF values (0.365 ± 0.201 mL/g/min at 1.5 T and 0.221 ± 0.128 mL/g/min at 3.0 T) did not demonstrate statistically significant differences between the two field strengths (*P *= NS). No significant difference was found between the two observers in terms of calculated MBF values at 1.5 T or 3.0 T (*P *= NS), although a slightly higher average MBF was obtained by the second observer at both *B*_0 _strengths (1.198 ± 0.205 mL/g/min at 1.5 T and 1.211 ± 0.281 mL/g/min at 3.0 T). Figure 5 shows the Bland-Altman analysis and demonstrates a trend toward less variation in the calculated MBF values by the two observers at 3.0 T as compared to 1.5 T.

### Error simulations

The relationship of the absolute error of the calculated MBF (in percentages) with the SNR of the images (35.60 at 1.5 T and 79.01 at 3.0 T) was determined with the error simulations. The data were obtained using the *T*_1 _values at 1.5 T. When the SNR is lower than 250 (for all 1.5 T and 3.0 T MRI systems), the error of the MBF is larger than 10% (10–50%). The repeatability of the MBF measurements in this simulation was found to be 0.127 for 1.5 T and 0.078 for 3.0 T (*P *= NS), whereas the error of the MBF was 13.9 ± 7.1% at 1.5 T and 5.4 ± 4.5% at 3.0 T (*P *= NS).

## Discussion

The primary goal of this study was to compare the accuracy and repeatability of myocardial perfusion data obtained at 3.0 T to that at 1.5 T using an ASL technique in normal human subjects. This ASL technique was previously validated at 1.5 T in a canine model [2].

The gold standard in human myocardial perfusion determination is PET, and ^13^N-ammonia is considered the tracer of choice, over ^15^O-water [21]. In the most recent ^13^N-ammonia PET study, Schepis, *et al*. found the MBF in resting human subjects to be in the range of 0.81 ± 0.28 to 0.84 ± 0.25 mL/g/min, depending on the PET method used [22]. Additionally, Rahimtoola compiled eight ^13^N-ammonia PET studies from the mid-1990's and found the range of MBF values (± 2 SD) in normal subjects to be approximately 0.40 to 1.50 mL/g/min [4]. In recent years, first-pass perfusion has been the primary CMR method used for the determination of myocardial perfusion. Using this technique, Hsu, *et al*. found the perfusion rate in resting human subjects to be 1.02 ± 0.22 mL/g/min [23]. Thus, our perfusion data at both field strengths compare favorably with the data obtained with ^13^N-ammonia PET and first-pass CMR.

Secondly, as anticipated, no significant difference was found between the myocardial perfusion data at the two different field strengths or between the perfusion data obtained by the two independent observers. The simulation results suggested the same finding; while the accuracy of perfusion data from the simulation at 3.0 T is somewhat better than that at 1.5 T (as 3.0 T displayed a 61% reduction in error), this difference was not found to be statistically significant. Additionally, less variation was observed in the perfusion data from the two observers at 3.0 T as compared to 1.5 T. Concordantly, the repeatability of MBF measurements at 3.0 T improved to 0.21 from 0.37 at 1.5 T, although these differences were not statistically significant. This is an improvement of approximately 43%; similar to the simulation result of a 38% improvement (*P *= NS). It should be noted that the absolute values of repeatability are much smaller than the measured ones, suggesting other errors contribute significantly, in addition to the effect of SNR. These errors may include incompletely labeled spins [24], transition time error [25], and imperfect slice profiles of data acquisition RF pulses [26]. Our simplified simulation accounted for none of these, and therefore, further investigations are needed to incorporate the effects of these error sources.

### Limitations

The major limitation of the ASL technique remains high sensitivity to motion. The requirement of ECG-gating and breath-holding for the duration of each series is not completely effective in eliminating cardiac and respiratory motion. Additionally, patient movement between the two back-to-back series introduced potential perfusion quantification error associated with inter-acquisition motion since the selective and non-selective scans were performed sequentially. All of these types of motion can lead to blurring and image distortion. In terms of the future application of this technique to patients, these motion types will be significant because patients requiring perfusion analysis will likely suffer from a myriad of comorbidities. It might be unreasonable to request a breath-hold of nearly 20 seconds in some patients, such as those with severe cardiovascular or respiratory disease.

Another limitation is associated with the MBF equation that was used in this study. This equation uses differences in *T*_1 _values between the slice-selective and nonselective acquisitions, rather than differences in signal intensity values, to calculate MBF. Notably, Zhou and van Zijl have theorized that in FAIR techniques, *T*_1 _value differences between the slice-selective and nonselective acquisitions may be increased or decreased, depending on the transit time of the "tagged bolus," while the signal intensity difference is always decreased [27]. The unpredictability of the effect of transit time makes it impossible to control for this source of error when *T*_1 _value differences are used to calculate MBF. In addition to this, perfusion quantification using FAIR techniques is susceptible to many sources of error, including macrovascular flow [28-30], radiation damping [31], the longer *T*_1 _of arterial blood [28,32,33], and imperfect inversion profiles [34].

There are two main limitations unique to the use of a 3.0 T MRI system for these techniques. First, the specific absorption rate (SAR) quadruples with the doubling of the field strength from 1.5 T to 3.0 T. This often necessitates increased repetition times and, consequently, increased examination durations. Second, magnetic susceptibility artifacts are more prominent at 3.0 T, particularly at the boundary of myocardial tissue and lung air, as well as the boundary of the myocardium and ventricular blood. The ROI was drawn at the center of the myocardium to avoid these effects. Nonetheless, imperfect magnetic shimming might still lead to these artifacts in some large subjects at 3.0 T. This was not often observed with the 1.5 T system.

Finally, this study quantified myocardial perfusion at rest. Such measurement may be helpful to monitor the efficacy of medical therapy in patients with cardiovascular disease. The next step in the study of this technique is to evaluate its efficacy during adenosine- or dipyridamole-induced vasodilatation. This would facilitate the determination of myocardial perfusion reserve, a more important clinical parameter. A study including this parameter as an end-point would be likely to demonstrate a greater reduction in the magnitude of error from 1.5 T to 3.0 T because the percent error of perfusion reserve is proportional to the percent error of myocardial perfusion during stress. The latter percent error would be greatly reduced during vasodilatation, when perfusion is three-to-four-fold greater than at rest [2].

## Conclusion

This work suggests that CMR ASL can be used to quantify myocardial perfusion at rest in humans with relative accuracy. However, there are several limitations to this technique, to the MBF equation that was used, and to the use of the error simulation to assess accuracy that must temper this suggestion. Furthermore, no significant difference in the use of 1.5 T and 3.0 T systems, in terms of the accuracy and repeatability of myocardial perfusion quantification, was found. However, 3.0 T did demonstrate a significantly higher SNR that yielded slightly improved repeatability, slightly less variation in perfusion data, and more uniform signals in perfusion maps. With continued success, this technique could be applied to patients with significant perfusion defects and eventually become part of the standard clinical work-up of patients with ischemic heart disease.

## Abbreviations

ASL: arterial spin labeling; CMR: cardiovascular magnetic resonance; ECG: electrocardiogram; FAIR: flow-sensitive alternating inversion recovery; GE: gradient echo; IR: inversion recovery; LV: left ventricle; MBF: myocardial blood flow; NonS: non-slice-selective prepared sequence; PET: positron emission tomography; ROI: region of interest; Sel: slice-selective prepared sequence; SNR: signal-to-noise ratio; SSFP: steady-state free precession; TI: inversion time.

## Competing interests

The authors declare that they have no competing interests.

## Authors' contributions

BEN participated in the study design, analyzed the data, and drafted the manuscript. KSM participated in the study design, carried out the computer simulations, and analyzed the data. HZ and SR coordinated subject recruitment, carried out the CMR examinations, and analyzed the early data. PKW, RJG, and JZ conceived of the study, participated in its design, and supervised all aspects of the study.
